# Celecoxib prevents malignant progression of smoking-induced lung tumors via suppression of the COX-2/PGE_2_ signaling pathway in mice

**DOI:** 10.3389/fimmu.2025.1557790

**Published:** 2025-03-19

**Authors:** Kaori Sakurai, Shotaro Chubachi, Jun Miyata, Junko Hamamoto, Tatsuro Naganuma, Takashi Shimada, Shiro Otake, Shingo Nakayama, Hidehiro Irie, Akihiro Tsutsumi, Naofumi Kameyama, Ahmed E. Hegab, Masayuki Shimoda, Hideki Terai, Hiroyuki Yasuda, Yae Kanai, Makoto Arita, Koichi Fukunaga

**Affiliations:** ^1^ Division of Pulmonary Medicine, Department of Internal Medicine, Keio University School of Medicine, Tokyo, Japan; ^2^ Division of Physiological Chemistry and Metabolism, Graduate School of Pharmaceutical Sciences, Keio University, Tokyo, Japan; ^3^ Laboratory of Biochemistry, Faculty of Pharmaceutical Sciences, Hokkaido University, Sapporo, Japan; ^4^ Medical Education Center, School of Medicine, International University of Health and Welfare, Narita, Japan; ^5^ Department of Pathology, Keio University School of Medicine, Tokyo, Japan; ^6^ Department of Pathology, The Jikei University School of Medicine, Tokyo, Japan; ^7^ Laboratory for Metabolomics, RIKEN Center for Integrative Medical Sciences (IMS), Yokohama, Japan; ^8^ Cellular and Molecular Epigenetics Laboratory, Graduate School of Medical Life Science, Yokohama City University, Yokohama, Japan; ^9^ Human Biology-Microbiome-Quantum Research Center (WPI-Bio2Q), Keio University, Tokyo, Japan

**Keywords:** chronic obstructive pulmonary disease, lung neoplasms, cyclooxygenase 2 inhibitor, smoking, prostaglandin E_2_, chemoprevention

## Abstract

**Introduction:**

Lung cancer is characterized by a poor prognosis and is a significant comorbidity of chronic obstructive pulmonary disease (COPD). Therefore, effective chemopreventive agents are warranted. We evaluated the effects of the cyclooxygenase-2 (COX-2) inhibitor celecoxib on the prevention of lung-carcinoma development using an intermittent smoking-induced lung-carcinoma mouse model. Additionally, we explored COX-2’s role in lipid metabolism.

**Methods:**

Male A/J mice were exposed to sham air or mainstream cigarette smoke for 20 weeks. Vehicle or celecoxib was administered via intragastric feeding once daily. Lung tissues were analyzed for tumor nodules and emphysema; the bronchoalveolar lavage fluid was collected for cell counting. COX-2 expression was measured using real-time polymerase chain reaction and western blotting; lipidomic analysis was conducted using liquid chromatography-tandem mass spectrometry. Cell proliferation and colony-forming assays were performed on LA-4 cells to assess the effects of prostaglandins and COX-2 inhibitors.

**Results:**

Intermittent smoking exposure increased lung adenomas, adenocarcinomas, and COX-2 expression. Lung adenomas were characterized by abundant COX-2-positive cells. Celecoxib reduced intermittent smoking-induced inflammation, emphysema, and cell counts in the bronchoalveolar lavage fluid and decreased the incidence of lung adenocarcinomas, whereas the total number of observed lung tumors was unchanged. Celecoxib markedly suppressed single-smoke-induced prostaglandin E2 (PGE_2_) production in the airway. PGE_2_ increased LA-4 cell viability via the EP4 receptor and promoted colony formation.

**Discussion:**

Celecoxib effectively inhibited lung-carcinoma development, inflammation, and emphysema, demonstrating the potential for chemoprevention in smokers and patients with COPD. Further studies on EP4 inhibitors for the prevention of emphysema and lung cancer are warranted.

## 1 Introduction

Lung cancer is a notable comorbidity of chronic obstructive pulmonary disease (COPD), accounting for 21–33% of COPD-related deaths (1, 2). Lung cancer associated with COPD has a poorer prognosis than that without COPD (3). Additionally, it is more likely to cause perioperative complications and treatment-related side effects (4). Therefore, there is a need for chemoprevention of lung cancer in smokers and patients with COPD. Despite numerous studies, no prophylactic agent has been established (5).

Smoking is a common risk factor for COPD and lung cancer. Previous studies, including our own, have shown that emphysema, an irreversible structural destruction, is a significant risk factor for lung-cancer development in patients with COPD (6, 7). It has been postulated that emphysema and lung cancer share a common pathogenesis, including genetic abnormalities, cell cycle abnormalities, chronic inflammation, cytokines, and a protease-antiprotease imbalance (8). Smoking-induced chronic inflammation is a major cause of carcinogenesis (9). Chronic inflammation induces alveolar destruction and emphysema through direct damage to the alveolar wall and indirect damage via proteases such as matrix metalloproteinases (10). Additionally, damage to the alveolar epithelium contributes to DNA damage, gene mutations, and the accumulation of epigenetic abnormalities, leading to the development of lung cancer (11). Chronic inflammation also promotes the growth and progression of existing tumors by enhancing cell proliferation, increasing resistance to apoptosis, and exerting immunomodulatory effects (11).

Cyclooxygenase (COX) is an enzyme responsible for the biosynthesis of prostanoids. Arachidonic acid (AA), an unsaturated fatty acid, is a substrate for COX and is converted into lipid mediators such as prostaglandins, prostacyclins, and thromboxanes (12). Two structurally distinct forms of the cyclooxygenase enzymes (COX-1 and COX-2) have been identified. COX-1 is a housekeeping enzyme responsible for maintaining the basal prostanoid levels, which are crucial for tissue homeostasis. In contrast, COX-2 is typically absent in most cells but is strongly induced at sites of inflammation and during tumor progression (12). COX-2 is also reported to be upregulated by smoking exposure and is considered a key enzyme in smoking-induced inflammation in the lungs (13, 14). In a previous study using a rat smoking model, COX-2 inhibitors suppressed intrapulmonary inflammation and inhibited the development of emphysema (15).

In recent years, lipidomic analysis has proven useful for comprehensively evaluating the dynamics of lipid metabolism (16, 17). Lipidomic analysis of a previous smoking-exposed mouse model indicated that smoking activated fatty acid metabolism in the COX pathway (18). However, no previous studies have examined the COX-2 dependence on the dynamics of fatty acid metabolism in smoking-exposed lungs or its functional significance.

COX-2 has multifaceted effects on tumors, including promoting growth, invasion, and metastasis (19). Chronic inflammation has been reported to contribute to tumor initiation (20). While COX-2 inhibitors have been associated with tumor progression, invasion, and metastasis (21), their role in suppressing tumor initiation, particularly in lung cancer, remains unclear. COX-2 has also been reported to be expressed in several cancers, including human lung cancer, and is linked to disease progression and poor prognosis (22, 23). Therefore, it has attracted attention as a target for chemoprevention of cancer (21). COX-2 inhibitors have been reported to prevent tumor development in chemically-induced lung-cancer models (24). Furthermore, knocking down the COX-2 gene inhibited K-ras-induced lung carcinogenesis (25). However, there are conflicting reports showing that although COX-2 inhibitor administration in a butylated hydroxytoluene-treated model improved intrapulmonary inflammation, it did not suppress lung tumors (26). To the best of our knowledge, no previous studies have examined whether COX-2 inhibitors are effective in preventing the development of lung tumors in a mouse model of smoking exposure.

We hypothesized that COX-2 inhibitors may have a preventive effect not only on smoking-induced emphysema but also on lung tumors. Animal models are crucial for developing drugs for the chemoprevention of lung cancer. Single-gene mutations and chemically-induced mouse models have been established for this purpose (24, 27, 28). However, these models have difficulty mimicking smoking-induced lung cancer. We established animal models that can examine emphysema and smoking-induced lung tumors caused by exposure to intermittent smoking (29). In this study, we aimed to i) examine whether COX-2 inhibitor administration successfully suppresses the development of smoking-induced lung cancer using our established mouse model and ii) examine the behavior of the COX metabolic pathway during smoking exposure by lipidomic analysis and elucidate the molecular mechanism of lung-cancer inhibition by examining the function of lipid metabolites through *in vitro* experiments.

## 2 Materials and methods

### 2.1 Mice

Male A/J mice (7–10 weeks old) were purchased from Sankyo Labo Service (Tokyo, Japan). The mice were housed in plastic cages under a 12:12-h light-dark cycle. The mice had 1 week of acclimatization prior to the investigation. All experimental procedures were in accordance with the National Institutes of Health guidelines and approved by the Laboratory Animal Center of Keio University School of Medicine.

### 2.2 NNK treatment, smoking exposure, and treatment groups

**Figure 1:**
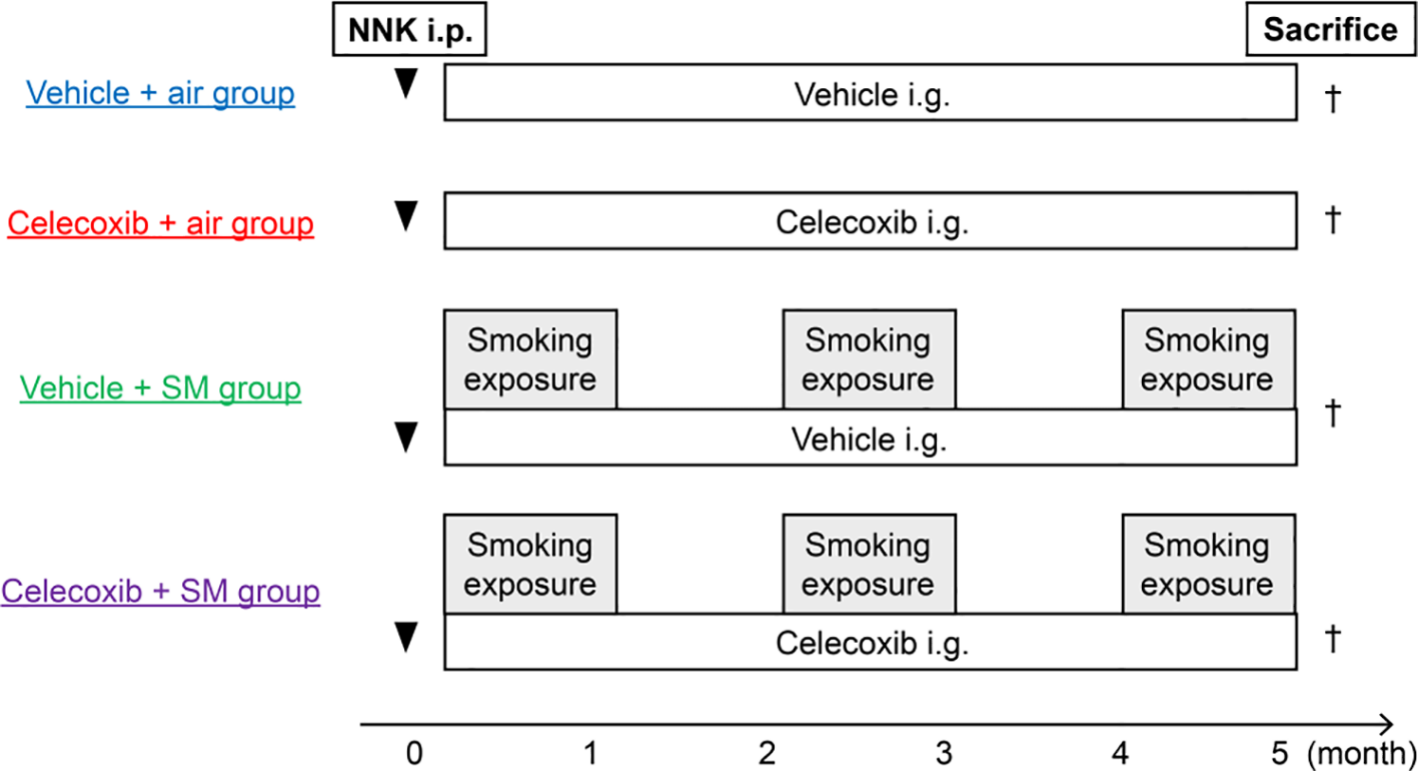
Study design and experimental protocols. Male A/J mice (7–10 weeks old) were exposed to sham air or cigarette smoke 2 weeks after NNK injection (i.p.). The vehicle or celecoxib was administered via intragastric feeding (i.g.). Tumor development and emphysema were analyzed at 20 weeks. SM, smoke.

After 2 weeks of intraperitoneal injection of a potent carcinogen (100 mg/kg), 4-(methylnitrosamino)-1-(3-pyridyl)-1-butanone (NNK) (Toronto Research Chemicals, Toronto, Ontario, Canada), mice were exposed to sham air or mainstream cigarette smoke generated from commercially available filtered cigarettes (Marlboro, 12 mg tar/1.0 mg nicotine, Philip Morris Inc., Richmond, VA, USA) for 1 h/day, 5 days/week for 20 weeks. Mice inhaled cigarette smoke through their noses, as previously reported (29). For this procedure, a cigarette smoke inhalation apparatus (SIS-CS system, Shibata Scientific Technology, Tokyo, Japan) which includes a cigarette smoke generator (SG-300) and an inhalation chamber, to which 20 body holders were set at a time, was used. The cigarette smoke was generated at a stroke volume of 15 mL and 10 puffs/min and diluted with compressed air, resulting in a total particulate matter concentration of 1,202 ± 196 mg/m^3^. Vehicle or celecoxib (BioVision, Milpitas, CA, USA) was administered (75 mg/kg) (30) via intragastric feeding once a day, 5 days/week for 20 week. Mice were divided into four groups: vehicle-plus-air (n = 15), celecoxib-plus-air (n = 17), vehicle-plus-smoke (n = 19), and celecoxib-plus-smoke (n = 21). The mice were sacrificed for analysis at 20 weeks (**Figure 1**).

### 2.3 Sampling of mouse lung tissue and bronchoalveolar lavage

The lungs were fixed by an intrabronchial infusion of 4% paraformaldehyde, with the pressure maintained at 25 cm of water. The lungs were removed, fixed, embedded in paraffin, and serially sectioned at 100-µm intervals. Tissue sections (6 µm) were stained with hematoxylin and eosin (H&E) for histopathological detection of tumor nodules, as previously reported (29). In another subgroup of mice, the lungs were lavaged with 0.6 mL of phosphate-buffered saline three times. Total cell counts and cell differentials in the bronchoalveolar lavage fluid (BALF) were examined as previously described (31). The BALF supernatants were stored at -80°C for further analysis.

### 2.4 Morphometric assessment of emphysema

The mean linear intercept, a standard parameter of alveolar size, and the destructive index indicating alveolar destruction were measured in ten randomly selected fields per mouse (29).

### 2.5 Pathological assessment of lung tumors

Bronchial-alveolar proliferative lesions were pathologically diagnosed as hyperplasia, adenoma, or adenocarcinoma on the H&E-stained sections, according to published criteria (32), by a trained pathologist (M.S.). The criteria for adenoma included rounded to columnar epithelial proliferation lining the alveoli in a uniform pattern, minimal to no cellular atypia, absence of mitosis, preservation of the original alveolar structure, and lack of fibrovascular stroma or clusters of plump epithelial cells. The criteria for adenocarcinoma included increased cellular atypia, loss of alveolar structure, higher mitotic activity, increased cellularity, and a papillary growth pattern. Tumor incidence, multiplicity, and size were calculated as previously described (29). All sections were evaluated blindly.

### 2.6 Quantitative real-time polymerase chain reaction analysis

Total RNA was isolated from whole lungs using an RNeasy Mini Kit (Qiagen, Hilden, NRW, Germany). Total RNA was reverse-transcribed using the High-Capacity RNA-to-cDNA Kit (Thermo Fisher Scientific, Waltham, MA, USA), following the manufacturer’s protocol. Real-time quantitative PCR analysis was performed with SYBR Green assays on the QuantStudio 5 Real-Time PCR System (Thermo Fisher Scientific). Mouse β-actin was used as the endogenous control for normalization. The primers used for COX-2 amplification were 5′-GGCGCAGTTTATGTTGTCTGT-3′ (forward) and 5′-CAAGACAGATCATAAGCGAGGA-3′ (reverse). The primers used for β-actin amplification were 5′-GGCTGTATTCCCCTCCATCG-3′ (forward) and 5′-CCAGTTGGTAACAATGCCATGT-3′ (reverse). Relative expression levels were calculated using the delta-delta Ct method.

### 2.7 Extraction of protein and western blot analysis

Briefly, the lung samples were homogenized in radio-immunoprecipitation assay buffer (Thermo Fisher Scientific). The protein concentration was quantified using a bicinchoninic acid assay, and equal amounts of protein (30 µg per lane) were loaded onto SDS-polyacrylamide gels and electrophoretically transferred to polyvinylidene fluoride membranes. The membranes were incubated overnight with primary antibodies at 4°C, followed by incubation with secondary antibodies for 1h. Anti-cyclooxygenase-1 antibody (Abcam, Cambridge, UK, ab109025, 1:1000 dilution), anti-cyclooxygenase-2 antibody (Abcam, ab15191, 1:1000 dilution), and anti-β-actin antibody (Sigma Aldrich, St. Louis, MO, USA, A5441, 1:5000 dilution) were used for the assessment. Immunoreactive proteins were visualized using the LumiGLO reagent and peroxide (Cell Signaling Technology, Danvers, MA, USA). Specific bands were captured using an LAS 4000 Mini System (GE Healthcare Life Sciences, Chicago, IL, USA). Relative protein expression was quantitated by densitometry (Image Quant TL software, GE Healthcare Life Sciences) and normalized against β-actin levels.

### 2.8 Immunofluorescence and immunohistochemical analysis

Immunofluorescence analyses were conducted on the vehicle-plus-smoke group. The lung sections were deparaffinized in xylene and rehydrated through a graded ethanol series. The primary antibody used was anti-COX-2 (GeneTex, Irvine, CA, USA, GTX15839, 1:100 dilution), and cells were counterstained with DAPI (Vector Laboratories, Burlingame, CA, USA). Fluorescence images were captured using a fluorescence microscope.

The vehicle-plus-smoke and celecoxib-plus-smoke groups were immunohistochemically analyzed. The primary antibody used was anti-PCNA (Dako, Glostrup, Denmark, M0879, 1:200 dilution), and cells were counterstained with hematoxylin.

### 2.9 Enzyme-linked immunosorbent assay

PGE_2_ levels in BALF were measured using an enzyme-linked immunosorbent assay (ELISA) kit (Cayman Chemical, Ann Arbor, MI, USA, 514010).

### 2.10 Targeted liquid chromatography-tandem mass spectrometry–based lipidomics

Lipidomic analysis was performed as previously described (33). BALF samples (900 μL) were dissolved in methanol. Deuterated internal standards (1 ng of eicosapentaenoic acid-d5, 15-hydroxyeicosatetraenoic acid (HETE)-d8, leukotriene B4-d4, PGE_2_-d4, and thromboxane B2-d4) were added to the methanolic extract. The samples were kept overnight at −30°C. The methanolic extract was then diluted in water. After centrifugation, the supernatant was subjected to solid-phase extraction using a MonoSpin C18-AX cartridge (GL Sciences, Tokyo, Japan). The column was eluted with 90% methanol (containing 2% acetic acid). The resulting solutions were then dried under nitrogen and concentrated. For LC-MS/MS analysis, we used a triple-quadrupole mass spectrometer (LCMS-8060; Shimadzu, Kyoto, Japan) attached to an ACQUITY UPLC BEH C18 column (1.0 × 150 mm, 1.7-μm particle size; Waters, Milford, MA, USA). The samples were eluted in a mobile phase consisting of water/acetate (100:0.1, v/v) and acetonitrile/methanol (4:1, v/v). Polyunsaturated fatty acid metabolites were analyzed in negative ion mode and quantified using multiple reaction monitoring for identification (LabSolutions Insight LCMS software, Shimadzu). Calibration curves between 0.1 and 100 pg and retention times for each compound were determined using synthetic standards. Quantification was performed using the calibration curves for each standard.

### 2.11 Cell line

The murine lung adenoma cell line LA-4 (ATCC, Manassas, VA, USA, CCL-196, purchased in December 2021) was grown in Ham’s F-12K (Kaighn’s) medium (Thermo Fisher Scientific) supplemented with 15% fetal bovine serum (Sigma Aldrich) and 1% penicillin-streptomycin at 37°C in a humidified 5% CO_2_ incubator, following the supplier’s protocol. Only early-passage cells were used for the experiments. The cell line was confirmed to be mycoplasma-free, with the last test conducted in September 2024 using the Lonza MycoAlert Mycoplasma Detection Kit (Lonza, Basel, Switzerland, LT07-118).

### 2.12 Cell proliferation assay

The MTS proliferation assay was conducted using the Cell Titer 96 AQueous One Solution Assay kit (Promega, Madison, WI, USA, G3581), according to the manufacturer’s protocol. Briefly, 5×10^3^ cells/well were seeded into 96-well plates and incubated for 24 h to allow cell adhesion.

1. Cells were treated with various concentrations of PGD_2_, PGE_2_, PGF_2α_, and 12- hydroxyheptadecatrienoic acid (HHTrE) (Cayman Chemical, 12010; 14010; 16010; 34590).

2. Cells were treated with EP receptor antagonists (50 nM of EP1 antagonist, 3 μM of EP2 antagonist, 0.1 μM of EP3 antagonist, and 1 μM of EP4 antagonist (Cayman Chemical, ONO-8711, 14070; TG11-77, 30188; L-826,266, 18538; ONO-AE3-208,14522) for 30 min before adding 1 μM of PGE_2_.

Control cells were treated with the same concentration of the vehicle, dimethyl sulfoxide (Wako, Osaka, Japan, 046-21981). After 24 h of treatment, absorbance was measured at 490 nm.

All experiments were performed at least three times, and the representative data are shown.

### 2.13 Soft agar colony-forming assay

Cells were seeded (1×10^4^ cell/well) in 0.33% low-melting-point agarose in Ham’s F-12K (Kaighn’s) medium in the presence or absence of PGE_2_ and layered onto 0.5% agarose in Ham’s F-12K (Kaighn’s) medium. The cell dishes were maintained in a culture incubator for 3 months. During this incubation period, 0.3 mL of fresh medium, with or without PGE_2_ (1 μM), was added twice weekly. Experiments were performed in triplicate on six-well plates and repeated three times. Representative photographs of colony samples were obtained. The colonies formed and total colony area were then counted using the ImageJ software (34). The colonies of >150 μm in diameter were counted.

### 2.14 Statistical analysis

The data are expressed as mean ± SE. The data were analyzed using Student’s t-test or one-way analysis of variance (ANOVA), followed by Tukey-Kramer’s *post hoc* test. Categorical data were analyzed using the χ^2^ test. All data were analyzed using JMP Pro version 17 (SAS Institute, Cary, NC, USA). All p-values were two-sided, and statistical significance was set at p < 0.05.

## 3 Results

### 3.1 Intermittent smoking exposure increases lung tumor development and COX-2 expression

**Figure 2:**
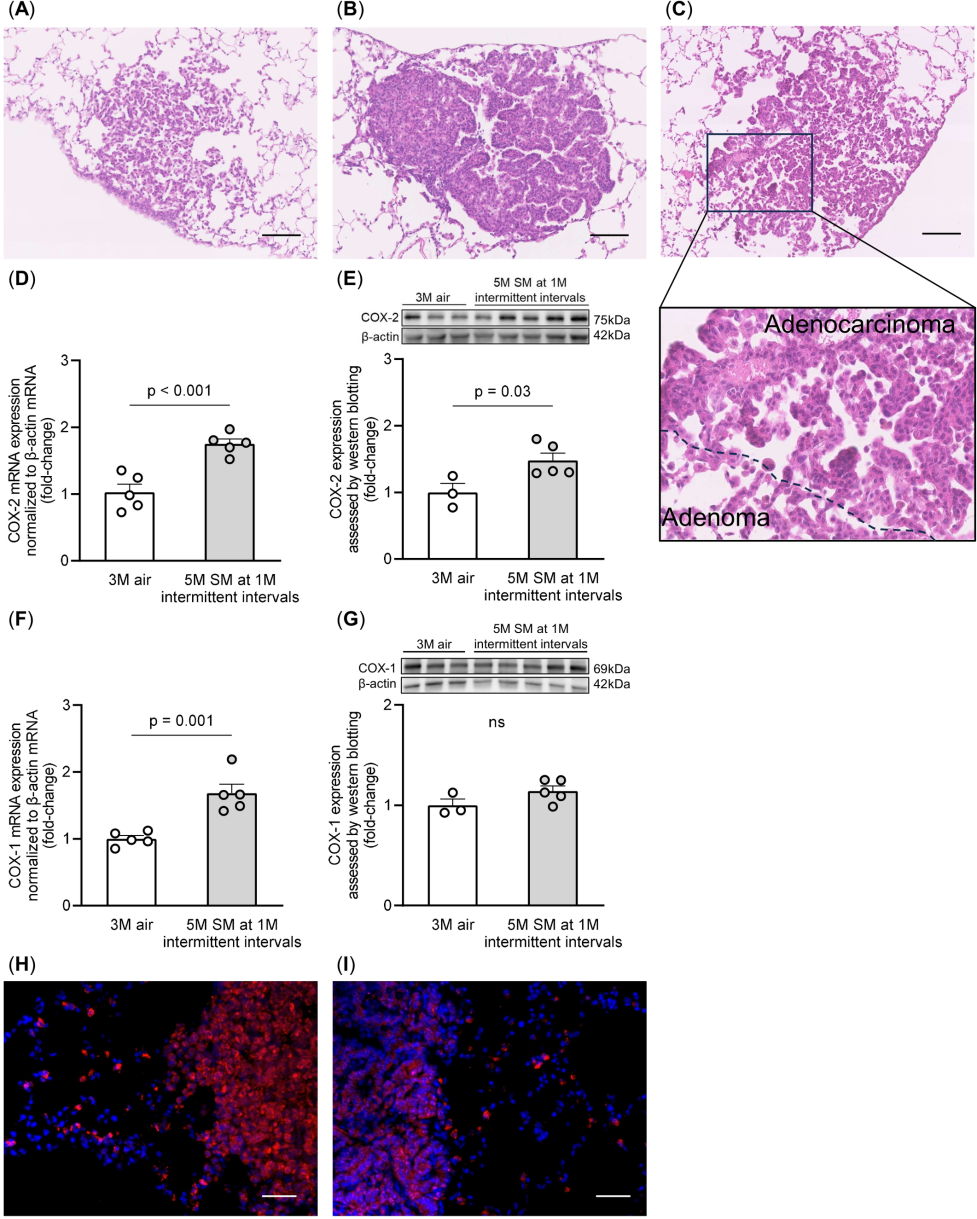
Morphological findings from lung tumors and COX-2 expression in the lung tissues. Representative images of **(A)** lung adenoma, **(B)** lung adenocarcinoma, and **(C)** mixed lung adenoma and adenocarcinoma within the same tumor, stained with hematoxylin and eosin, are shown. Scale bars: 100 μm. **(D, E)** COX-2 mRNA and protein levels were measured by RT-qPCR and western blotting, respectively, normalized to β-actin levels. **(F, G)** COX-1 mRNA and protein levels were measured by RT-qPCR and western blotting, respectively, normalized to β-actin levels. The data are shown as mean ± SE. Statistical analysis was performed with a Student’s t-test. The uncropped gels and blots are provided in (**Supplementary Figure 3**). **(H, I)** COX-2 expression (red) in the lung adenoma **(H)** and adenocarcinoma **(I)** cells was examined by immunostaining. Tissues were counterstained with DAPI (blue). Scale bars: 50 μm. SM, smoke; 1M, 1 month; 3M, 3 months; 5M, 5 months.

Intermittent smoking exposure induced the formation of lung adenomas (**Figure 2A**) and adenocarcinomas (**Figure 2B**), as previously reported (29). Some tumors exhibited a mixture of lung adenoma and adenocarcinoma features within the same lung tumor (**Figure 2C**). To assess COX-2 expression, we performed quantitative RT-PCR analysis and immunoblotting of lung samples from non-smoking and intermittent smoking-exposed groups. The lung samples used in this study were previously obtained from mice exposed to intermittent smoking (29). The mRNA and protein expression levels of COX-2 were higher in the intermittent smoking-exposed lungs than in the non-smoking group (**Figures 2D, E**). However, in the case of COX-1, mRNA levels were elevated in the lungs exposed to intermittent smoking, whereas no significant difference was observed at the protein level (**Figures 2F, G**). Immunostaining revealed a higher abundance of COX-2-positive cells in the lungs of the intermittent smoking-exposed group than in those of the non-smoking group, with a greater prevalence in lung adenomas (**Figure 2H**) than in lung adenocarcinomas (**Figure 2I**). These findings suggest that intermittent smoking promotes COX-2 upregulation in early-stage lung tumors, particularly in adenomas, which may contribute to tumorigenesis.

### 3.2 Celecoxib suppresses inflammation and emphysema formation caused by intermittent smoking exposure

**Figure 3:**
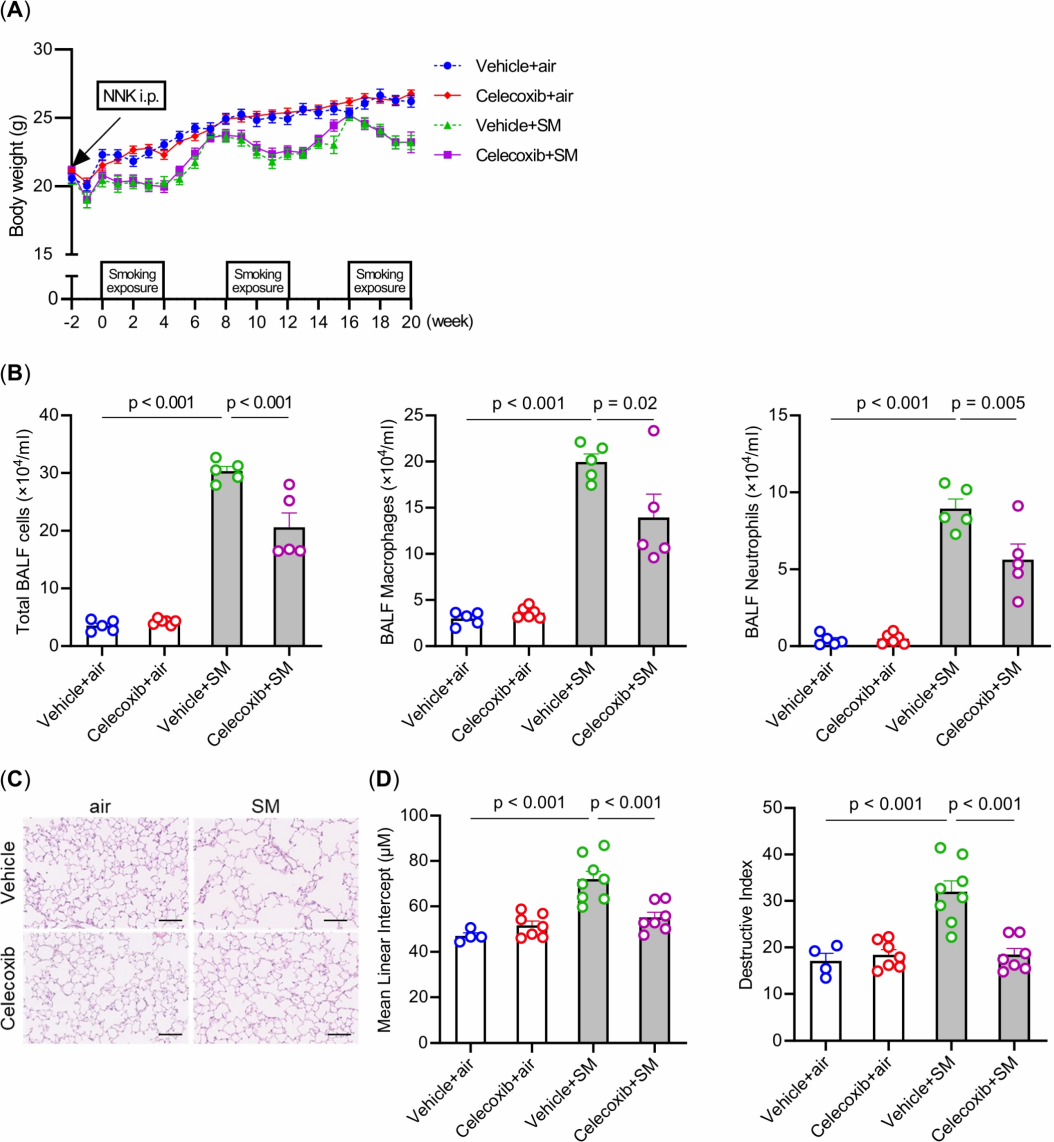
Effects of smoking exposure and celecoxib administration on body weight, inflammatory cells in the BALF, and emphysema in the lungs. **(A)** Changes in body weight over 5 months. **(B)** Total cell numbers and cell fractionation in BALF. **(C)** Histological assessment of emphysema in the lungs. Hematoxylin and eosin-stained lung sections are shown. Scale bars: 100 μm. **(D)** Comparison of mean linear intercept and destructive index. The data are shown as mean ± SE. Statistical analysis was performed with ANOVA, followed by *post hoc* analysis with the Tukey-Kramer test. Vehicle-plus-air group (blue); Celecoxib-plus-air group (red); Vehicle-plus-smoke group (green); Celecoxib-plus-smoke group (purple). SM, smoke.

Celecoxib did not affect body weight in either the air or intermittent smoking groups (**Figure 3A**). Celecoxib reduced the increase in total cells, macrophages, and neutrophils in BALF induced by intermittent smoking exposure (**Figure 3B**). Linear intercept and destructive index were significantly higher in the vehicle-plus-smoke group than in the vehicle-plus-air group. Additionally, they were significantly lower in the celecoxib-plus-smoke group than in the vehicle-plus-smoke group (**Figures 3C, D**). These results suggest that celecoxib mitigates intermittent smoking-induced lung inflammation and emphysematous changes, potentially contributing to its protective effects against smoking-related lung damage.

### 3.3 Celecoxib prevents intermittent smoking exposure-induced lung-cancer development

**Figure 4:**
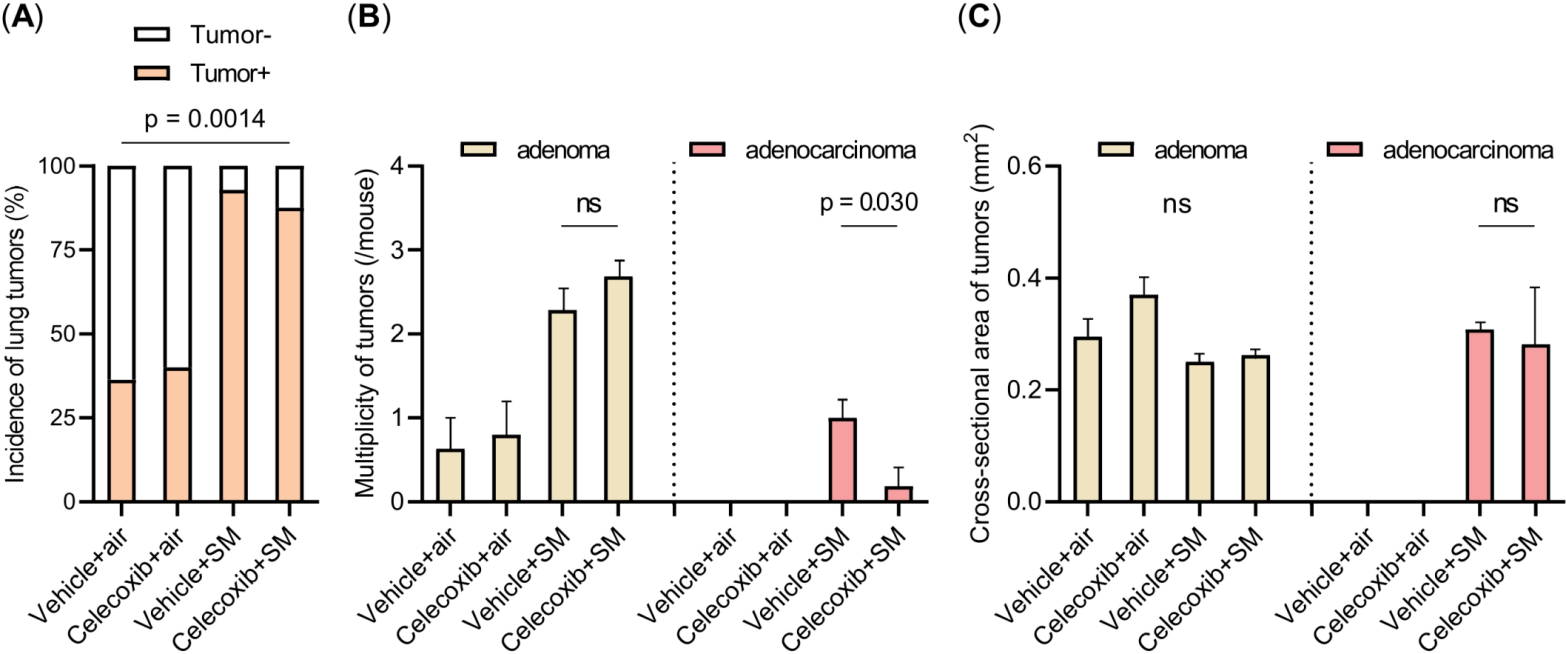
Incidence, multiplicity, and size of lung tumors. **(A–C)** The incidence **(A)**, multiplicity **(B)**, and size of lung tumors **(C)** are shown. The data are shown as mean ± SE. Statistical analysis of tumor incidence was performed with the χ^2^ test. Statistical analysis of the multiplicity and size of the tumor was performed with Student’s t-test or ANOVA, followed by *post hoc* analysis with the Tukey-Kramer test. ns, not significant; SM, smoke.

We compared the incidence, multiplicity, and size of lung tumors among the four groups (**Figures 4A–C**). The vehicle-plus-smoke group exhibited higher lung tumor incidence and multiplicity than the vehicle-plus-air group. However, the mean lung tumor size demonstrated no significant differences among the four groups. The celecoxib-plus-smoke group had fewer lung adenocarcinomas than the vehicle-plus-smoke group, even though the total numbers of whole lung tumors and lung adenomas were not significantly different. No significant differences in tumor incidence were observed between the two groups. These results may indicate that celecoxib administration prevented the progression from adenoma to adenocarcinoma. Next, we examined PCNA staining in adenomas and adenocarcinomas in celecoxib-treated and untreated groups. Both tumor types displayed PCNA-positive cells overall, with adenocarcinomas showing a higher prevalence than adenomas (**Supplementary Figure 1**). Celecoxib appeared to reduce the number of PCNA-positive cells in adenomas and adenocarcinomas. This reduction in PCNA expression suggests that celecoxib may suppress tumor cell proliferation, which could contribute to its inhibitory effect on adenoma-to-adenocarcinoma progression.

### 3.4 Effect of celecoxib on lipid mediators in bronchoalveolar lavage fluid under single smoking exposure

**Figure 5:**
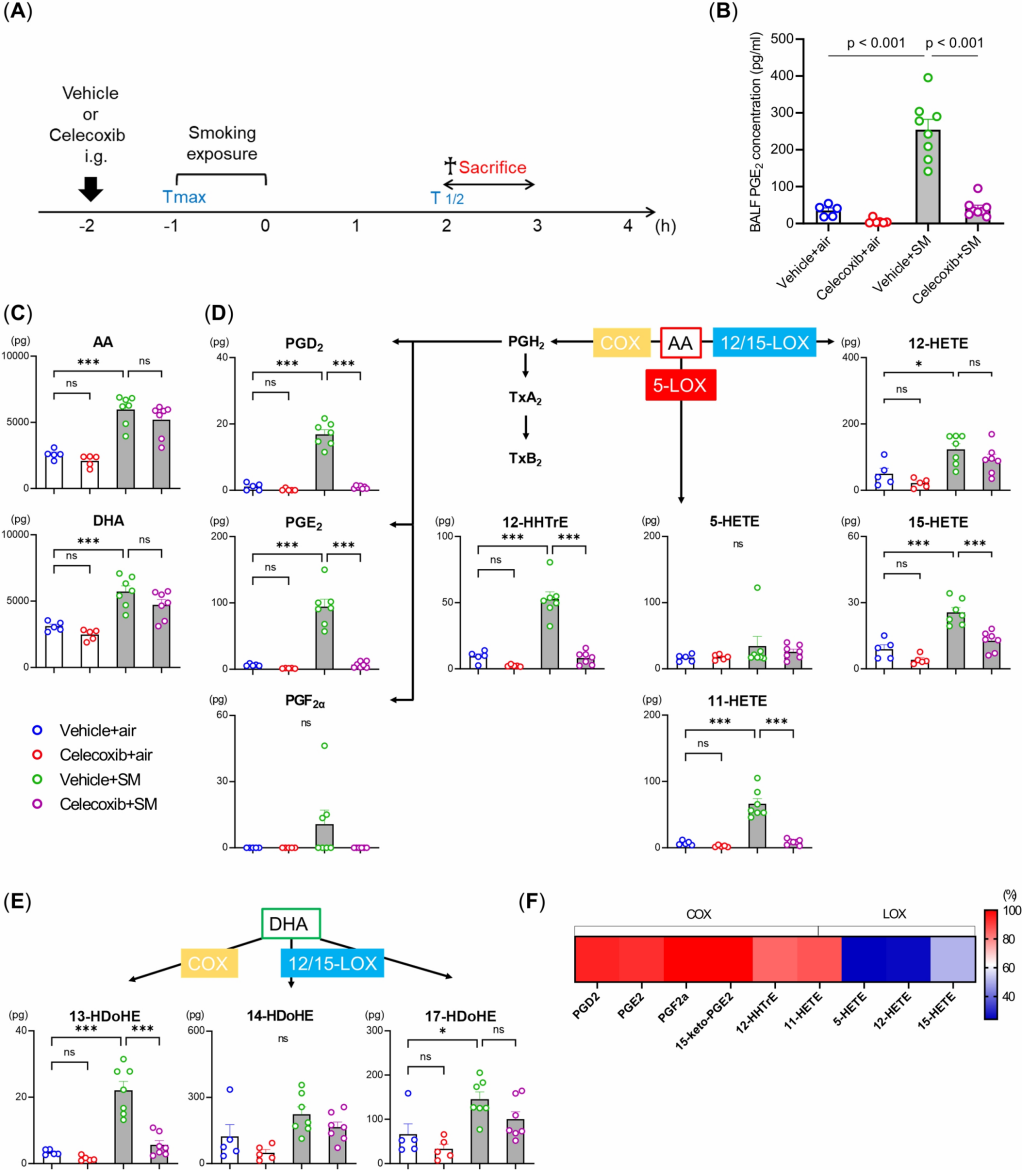
Effects of smoking exposure and celecoxib on lipid mediators in the BALF under single smoking exposure. **(A)** BALF samples from four experimental groups (with or without single smoking exposure or celecoxib) were collected as illustrated. h: hour. **(B)** Concentration of PGE_2_ levels measured by ELISA. **(C–E)** Lipidomic profiles of BALF after single smoking exposure. Lipidomic analysis revealed quantitative alterations of AA and DHA-derived metabolites via COX, 5-LOX, and 12/15-LOX, including prostaglandins (PG), 12-HHTrE, HETE, and HDoHE after single smoking exposure. The data are shown as mean ± SE (n = 5–7 per group). Statistical analysis was performed with ANOVA, followed by *post hoc* analysis with the Tukey-Kramer test. ns: not significant, *p < 0.05, ***p ≤ 0.001, SM, smoke. **(F)** Heat map of the inhibition percentages of celecoxib.

To evaluate fatty acid metabolism in the lungs in response to smoking exposure, fatty acid metabolites were quantitatively measured in BALF samples from the four experimental groups (with or without a single smoking exposure or celecoxib treatment) (**Figure 5A**). We used an ELISA kit to assess the production of PGE_2_, a major product of the enzymatic reaction catalyzed by COX-2. Celecoxib significantly suppressed the smoke-induced PGE_2_ production (**Figure 5B**). Subsequently, we conducted LC-MS/MS-based mediator lipidomic analysis to reveal the global alterations in fatty acid metabolites induced by a single smoke exposure and celecoxib. AA and docosahexaenoic acid (DHA) increased with a single smoking exposure. Among COX-mediated metabolites, PGD_2_, PGE_2_, PGF_2α_, 12-HHTrE, and 11-HETE produced from AA, along with 13- hydroxy docosahexaenoic acid (HDoHE) from DHA, were increased by single smoking exposure (**Figures 5C–E**). Among these, PGE_2_ was the most abundant (**Supplementary Figure 2**). Among the 12/15-lipoxygenase (LOX)-mediated metabolites, 12-HETE, and 15-HETE from AA as well as 17-HDoHE from DHA were also increased by a single smoking exposure (**Figures 5D, E**). However, their downstream specialized pro-resolving mediators (SPMs), such as protectins, maresins, resolvins, and lipoxins, were not detected in picogram-level quantities. The amount of 5-HETE, a 5-LOX-mediated metabolite derived from AA, was not altered by a single smoking exposure (**Figure 5D**).

Celecoxib administration significantly reduced the levels of COX-mediated metabolites without affecting the levels of AA and DHA (**Figures 5C–F**). The production of 12/15-LOX-mediated metabolites was also mildly reduced, though not as much as the COX metabolites (**Figures 5D–F**). These results demonstrate that smoking exposure activates fatty acid metabolism through COX, characterized by increased PGE_2_ production in the lungs, and that celecoxib selectively inhibits this process.

### 3.5 Proliferative and malignant effects of PGE_2_ on pulmonary adenoma cells

**Figure 6:**
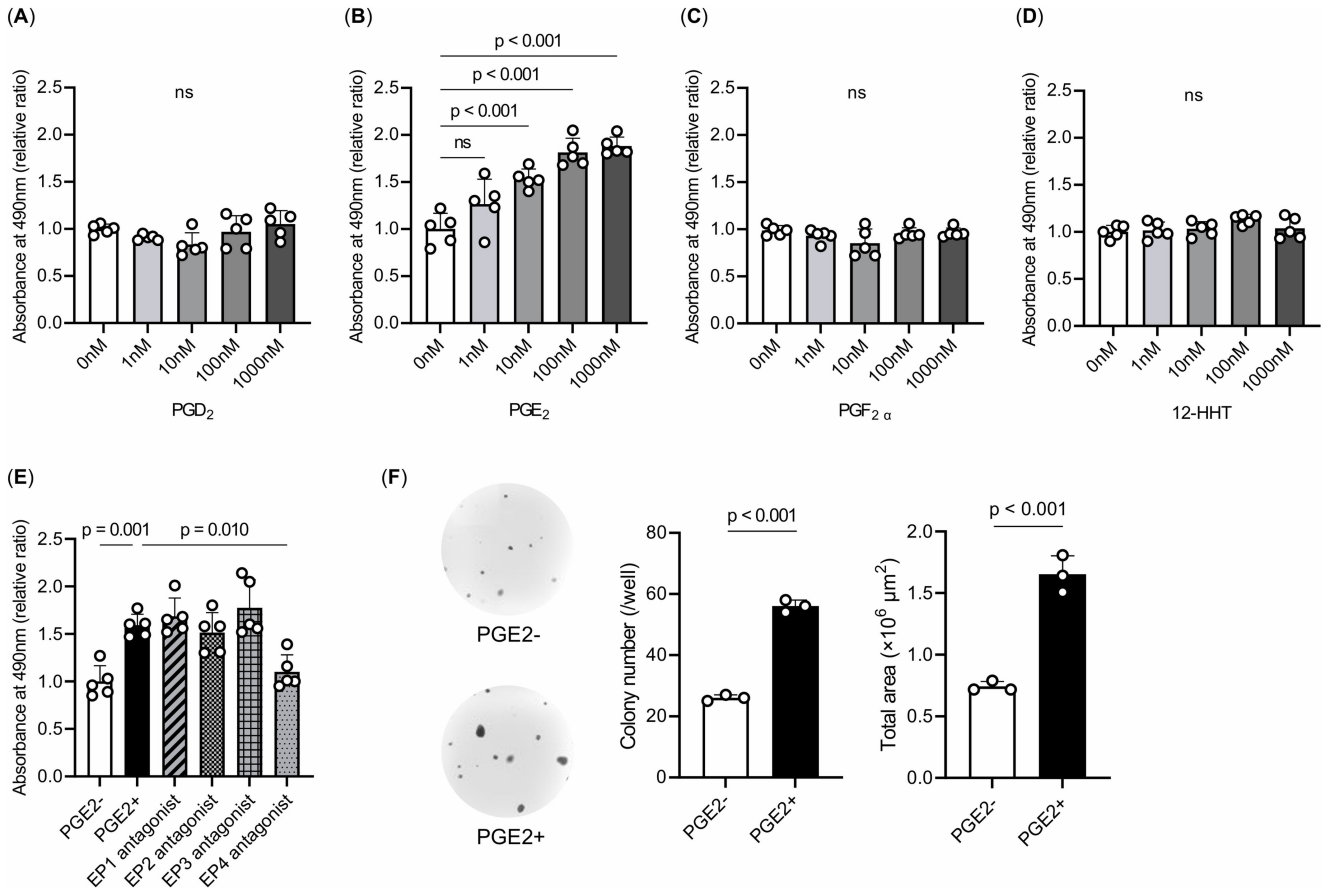
Effects of COX metabolites on cell proliferation and colony formation. **(A–E)** MTS assays. Treatments with **(A)** PGD_2_, **(B)** PGE_2_, **(C)** PGF_2α_, and **(D)** 12-HHTrE were evaluated. Only PGE_2_ increased cell viability in a concentration-dependent manner. **(E)** Treatments with EP1–4-specific antagonists. These experiments were repeated at least three times in quintuplicate, and representative data are shown. The data are presented as a fold increase over the vehicle-treated control. **(F)** Colony formation of LA-4 cells in soft agar. The colony numbers and total colony area were counted. PGE_2_ significantly increased colony formation in soft agar compared with the control group. Experiments were repeated three times in triplicate, and representative data and photographs are shown. Magnification, ×20. The data are shown as mean ± SE. Statistical analysis was performed with Student’s t-test or ANOVA, followed by *post hoc* analysis with the Tukey-Kramer test. ns, not significant.

We sought to verify the inhibitory effect of celecoxib on the transition from lung adenoma to adenocarcinoma, as suggested by our animal experiments, using the LA-4 cell line derived from mouse lung adenomas. We performed an MTS cell proliferation assay to investigate whether the COX metabolites elevated by a single smoking exposure and suppressed by celecoxib in the lipidomic analysis would enhance LA-4 cell viability. Treatments with PGD_2_, PGE_2_, PGF_2α_, and 12-HHTrE were evaluated. Only PGE_2_ increased cell viability in a concentration-dependent manner (**Figures 6A–D**). PGE_2_ binds to and activates four distinct receptor subtypes named EP1–4. We performed an MTS cell proliferation assay using ligand antagonists of the EP subtypes to elucidate the mechanisms underlying PGE_2_-mediated enhancement of cell viability. Treatment with an EP4 antagonist reduced cell viability and increased PGE_2_ in LA-4 cells (**Figure 6E**). These results indicate that PGE_2_ enhances cell viability via the EP4 receptor. Next, to investigate whether PGE_2_ enhances the malignant potential of lung adenoma cells, we examined their anchorage-independent growth in soft agar. LA-4 cells were cultured in soft agar either with or without PGE_2_. PGE_2_ significantly increased colony formation in soft agar compared to the control (**Figure 6F**). These findings suggest that PGE_2_, through activation of the EP4 receptor, not only promotes cell proliferation but also contribute to the malignant transformation of lung adenoma cells.

## 4 Discussion

To the best of our knowledge, this is the first study to elucidate the molecular mechanisms underlying the protective effects of celecoxib against smoking-induced emphysema and lung cancer. Although transgenic mouse models and single chemical-induced models are also used as models of lung cancer, the smoking-induced lung-cancer model is superior due to its more physiological relevance (35). The results of this study suggest that celecoxib may help prevent the development of smoking-induced lung cancer. The molecular mechanism underlying the effect of celecoxib involves the inhibition of the EP4-mediated PGE_2_ effects, which were enhanced by smoking exposure. These findings indicate that celecoxib may be clinically useful for preventing lung-cancer development in smokers and patients with emphysema and COPD. In cancer prevention, there are three key concepts: primary prevention, which aims to prevent tumor initiation; secondary prevention, which seeks to prevent the malignant progression of existing tumors; and tertiary prevention, which includes reducing or controlling the symptoms and morbidity of established cancer (36). This study demonstrated the preventive effect of celecoxib on lung cancer development. However, considering its role in preventing the transition from lung adenoma to adenocarcinoma, its clinical significance may be more aligned with secondary rather than primary prevention. Celecoxib may be beneficial for patients with pre-neoplastic lesions such as atypical adenomatous hyperplasia or post-surgical patients.

COX-2, typically absent in healthy cells, is strongly induced at sites of inflammation and during tumor progression (12). Here, we found that COX-2 expression was higher in lung specimens exposed to intermittent smoking than in those of the non-smoking group. This aligns with previous studies reporting that smoking exposure induces COX-2 expression in lung fibroblasts (14), neutrophils, and macrophages (13). In this study, celecoxib administration suppressed intrapulmonary inflammation and emphysema formation caused by intermittent smoking exposure. A previous study in rats reported that systemic exposure to smoking for 20 weeks followed by celecoxib administration suppressed NFκB-mediated intrapulmonary inflammation and emphysema (15). These results suggest that celecoxib may be effective in the treatment of emphysema caused by smoking, regardless of the smoking pattern. We administered celecoxib during the early prophylactic phase alongside smoking to improve smoking-induced emphysema. However, in human clinical practice, emphysema is typically treated only after it has significantly developed. In general, mouse models of smoking-induced emphysema differ from patients with COPD, as they typically exhibit only mild emphysema, corresponding to GOLD 1 and 2, and show minimal airway disease (37). Future studies are required to determine whether celecoxib ameliorates advanced emphysema and whether it is effective in inhibiting emphysema progression in patients with COPD.

Notably, celecoxib administration specifically reduced the multiplicity of lung adenocarcinomas, although there was no significant difference in the multiplicity of lung adenomas or whole lung tumors. In this study, celecoxib appeared to reduce the number of PCNA-positive cells in adenomas and adenocarcinomas. Furthermore, PGE_2_ increased LA-4 cell viability in a concentration-dependent manner. These results are consistent with previous reports indicating that celecoxib decreased the number of Ki-67-positive bronchial cells in former smokers (38). It is difficult to determine from our results whether celecoxib suppresses the development of lung adenocarcinoma by inhibiting *de novo* carcinogenesis or by preventing the progression from lung adenoma to adenocarcinoma. A previous study using chemically induced lung-cancer mouse models reported that the frequency of lung adenomas decreases while the proportion of adenocarcinomas increases over time (39). Although we were unable to evaluate tumors before 5 months, investigating different time points can provide further insights into the antitumor effects of celecoxib. Although this study did not perform quantitative analysis of PGE_2_ or other lipid mediators in lung adenomas and adenocarcinomas, further investigation is warranted to strengthen the association between fatty acid metabolism, including PGE_2_, and the transition from adenoma to adenocarcinoma. The adenoma-adenocarcinoma sequence is widely known to occur in colorectal cancer (40). In mice and humans, a stepwise transition from hyperplasia to adenocarcinoma has been postulated (41). Tumors with characteristics of lung adenoma and lung adenocarcinoma in lung specimens exposed to intermittent smoking suggest that the latter mechanism is the most likely underlying mechanism. Notably, COX-2 expression was higher in lung adenomas than in lung adenocarcinomas due to intermittent smoking exposure. In a human phase 2 trial, the combined effect of chemotherapy and celecoxib was more pronounced than that of chemotherapy alone in patients with high COX-2 expression in the tumor tissue (42). The high expression of COX-2 in adenomas may explain why COX-2 inhibitors were effective against them. Previous histological studies on lung cancer have reported that COX-2-positive tumors tend to be more common in smokers (43). COX-2 is also known to be expressed early in carcinogenesis and even in the relatively early stages of chemically-induced lung cancer in mice (44). Approximately 71.6% of human lung atypical adenomatous hyperplasia is positive for COX-2 by immunostaining (45). Based on these findings, COX-2 may play an important role in bridging the gap between precancerous lesions and invasive cancer.

In general, studying chemopreventive agents for cancer in humans is challenging because of the long time required to define targets and outcomes (5). Despite these challenges, celecoxib has been reported to inhibit bronchial epithelial growth and precancerous lesions in bronchoscopic specimens of former smokers treated with COX-2 inhibitors (38). However, it remains to be examined whether celecoxib is effective in patients with COPD and precancerous lesions. Ideal chemopreventive agents should be well-tolerated, inexpensive, and potentially useful for treating comorbid diseases in high-risk individuals. Celecoxib is widely used as an anti-inflammatory drug. Although no apparent side effects were observed in this study, large human studies have suggested the possibility of cardiovascular complications, such as myocardial infarction and stroke, associated with long-term high-dose celecoxib use (46, 47). However, the results of this study suggest that celecoxib may also be effective in inhibiting the progression of COPD and emphysema, thus offering great clinical promise. Further investigation is needed to assess the optimal dosage, duration of administration, and suitable patient populations. PGE_2_ is primarily formed through metabolism of arachidonic acid by cyclooxygenases and the terminal enzyme microsomal prostaglandin E synthase-1 (mPGES-1). Selective inhibition of downstream mPGES-1 to reduce only PGE_2_ biosynthesis is suggested as a safer therapeutic strategy (48). Mice lacking in mPGES-1 have slower growing tumors and decreased angiogenesis and metastasis (49). Future studies on the preventive effects of mPGES-1 inhibitors on emphysema and lung cancer are warranted.

This is the first study on the effects of celecoxib as a compound on fatty acid metabolism regulation in smoking-exposed lung tissue. In this study, COX metabolism, with PGE_2_ as the most abundant metabolite, was enhanced in airways exposed to single smoking events. PGE_2_ plays dual roles in the activation and regulation of inflammation. In the early stages, it promotes local vasodilation and the accumulation of neutrophils and macrophages, whereas in later stages, it suppresses both innate and antigen-specific immunity at multiple molecular and cellular levels. Its production is tightly regulated by epithelial cells, alveolar macrophages, and other immune cells in response to infection, injury, or inflammation (50). Celecoxib effectively inhibited the formation of these COX metabolites. This is consistent with previous studies reporting consistent increases in AA, PGD_2_, and PGE_2_ levels in the lungs of mice after long-term smoking for 2, 3, and 7 months (18). The suppression of the COX pathway and intrapulmonary inflammation observed in this study aligns with reports that antagonism of PGD_2_ receptors led to the suppression of intrapulmonary inflammation in a mouse model of smoking (51). Additionally, PGE_2_ induced intrapulmonary inflammation and lung fibrosis and was associated with the pathogenesis of COPD (52), and smokers had higher levels of urinary PGE_2_ metabolites than nonsmokers (53). Thus, COX metabolism may be an important therapeutic target for smoking-related intrapulmonary inflammation and emphysema. Of interest, 12/15-LOX metabolism was also reduced in celecoxib-treated mice. However, as this study utilized BALF for lipidomic analysis, the levels of detected mediators were lower than in lung tissues in previous work (54). As a result, only precursors of pro-resolving mediators (17-HDoHE, 14-HDoHE, and 13-HDoHE) were detected. Some prostaglandins and leukotrienes are typically pro-inflammatory lipid mediators, others have an anti-inflammatory effect. SPMs such as lipoxins and resolvins which are biosynthesized via the 5-LOX plus 12/15-LOX pathway, have been suggested to play important roles in the resolution of inflammation (55). Previous reports have demonstrated a relationship between 15-LOX and COX-2. Acetylated COX-2 contributes to the production of 15(R)-HETE in endothelial cells. In addition, PGE_2_ switches 5-LOX metabolism and LTB_4_ synthesis to 15-LOX metabolism and LXA_4_ synthesis in human neutrophils. These findings partially explain the relationship between COX-2 inhibition and the impairment of 15-LOX metabolism (56). Collectively, this raises the possibility that SPMs could play a role in the regulation of cancer progression, which warrants further investigation.

A previous study reported that lung-cancer tissues from cigarette smokers exhibited elevated levels of PGE_2_ compared to those from nonsmokers with lung cancer (57), suggesting that PGE_2_ is a lipid metabolite present in smoking-induced lung tumors. In this study, among COX-dependent metabolites, only PGE_2_ enhanced the viability of lung adenoma cells in a concentration-dependent manner, according to the MTS assay. The addition of PGE_2_ enhanced the colony-forming ability of lung adenoma cells in the colony-forming assay, indicating *in vitro* tumorigenicity. The COX-2/PGE_2_ pathway has been reported to have various effects on tumors, including the promotion of angiogenesis, invasion, anti-apoptosis, and tumor immunosuppression (19, 22). Prostaglandins exert their biological effects in an autocrine or paracrine manner by binding to their respective cell surface receptors, which belong to the G protein-coupled receptor family. For PGE_2_, these receptors are designated as EP1, EP2, EP3, and EP4 (12). Each EP interacts with its unique G protein to activate particular downstream signaling pathways such as the protein kinase A pathway, β-catenin pathway, nuclear factor-kappa B pathway, and phosphatidylinositol 3-kinase/AKT pathway. These pathways play various roles in regulating biological behaviors (58). Activated EP1 can upregulate intracellular calcium ion concentrations; EP2 and EP4 receptors are associated with cyclic AMP stimulation and protein kinase A signaling through sequential activation of Gas and adenylyl cyclase; and EP3 is responsible for downregulating cyclic AMP levels (58). Previous studies have demonstrated that EP receptor subtypes (EP1, EP2, EP3, and EP4) contribute to lung-cancer progression (59–66), suggesting the potential therapeutic advantage of targeting PGE_2_ synthesis in the lungs rather than focusing on individual receptors. Additionally, previous *in vivo* studies have reported that EP4 is expressed during the oncogenic process of colorectal cancer and enhances the malignant potential of colorectal adenomas (67). Genetic and drug inhibition of EP4 can inhibit intestinal tumor growth (68). This is consistent with the fact that EP4-mediated signaling inhibited the growth potential of lung adenoma cell lines in this study. Furthermore, previous study on human lung-cancer specimens indicated that the intensity of EP4 expression is associated with a poor prognosis (69). EP4 inhibitors may have potential utility in preventing smoking-induced lung cancer. Further studies are warranted to investigate their effects using LA4 cell colony formation assays and an intermittent smoking exposure mouse model. Additionally, future research should explore the preventive effects of EP4 inhibitors on both emphysema and lung cancer.

In conclusion, in a mouse model of intermittent smoking-induced lung cancer, celecoxib administration suppressed intrapulmonary inflammation, emphysema, and the development of lung adenocarcinoma. Lipidomic and *in vitro* analyses revealed that celecoxib inhibits the malignant transformation of precancerous lesions and counteracts the EP4-mediated effects of PGE_2_. These findings suggest that therapeutic interventions targeting the COX-2 pathway may be useful for the chemoprevention of lung cancer in smokers and patients with COPD. Future studies are warranted to further explore these findings.

## Data Availability

The datasets presented in this study can be found in online repositories. The names of the repository/repositories and accession number(s) can be found below: LC-MS/MS data are available at the DROP Met section of the RIKEN PRIMe (http://prime.psc.riken.jp/menta.cgi/prime/drop_index) via the index DM0062.
