# Supplementary material for: Celecoxib prevents malignant progression of smoking-induced lung tumors via suppression of the COX-2/PGE2 signaling pathway in mice
Source: Front Immunol. 2025 Mar 19;16:1557790. doi: 10.3389/fimmu.2025.1557790 (PMC11961424; doi:10.3389/fimmu.2025.1557790)
Supplement: Supplementary file 1 [file DataSheet1.pdf]

Supplementary Material

1 Supplementary Figures

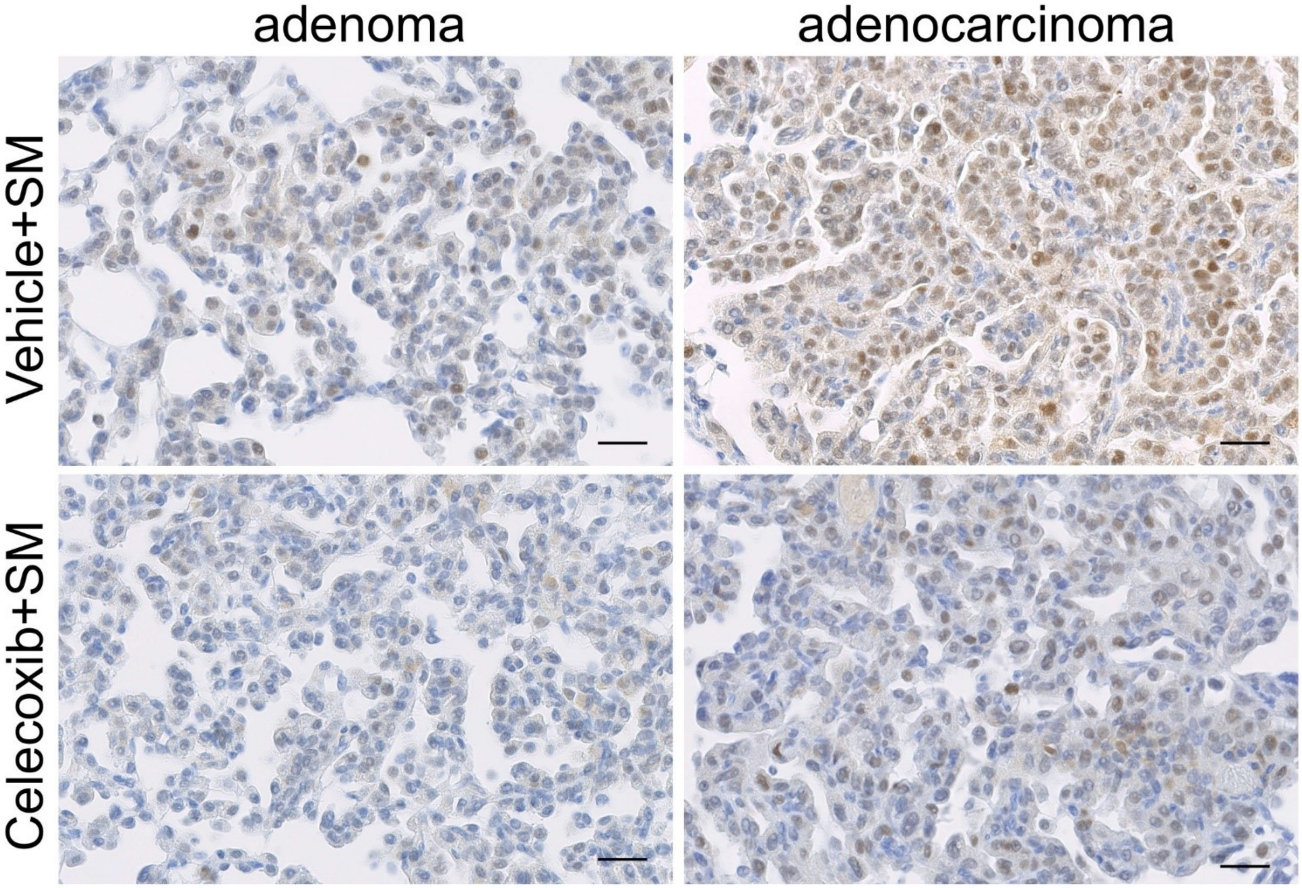

Supplementary Figure 1. PCNA staining (brown) in lung adenoma and adenocarcinoma from the vehicle-plus-smoke group and the celecoxib-plus-smoke group. Tissue sections were counterstained with hematoxylin. Scale bars: 25 µm.

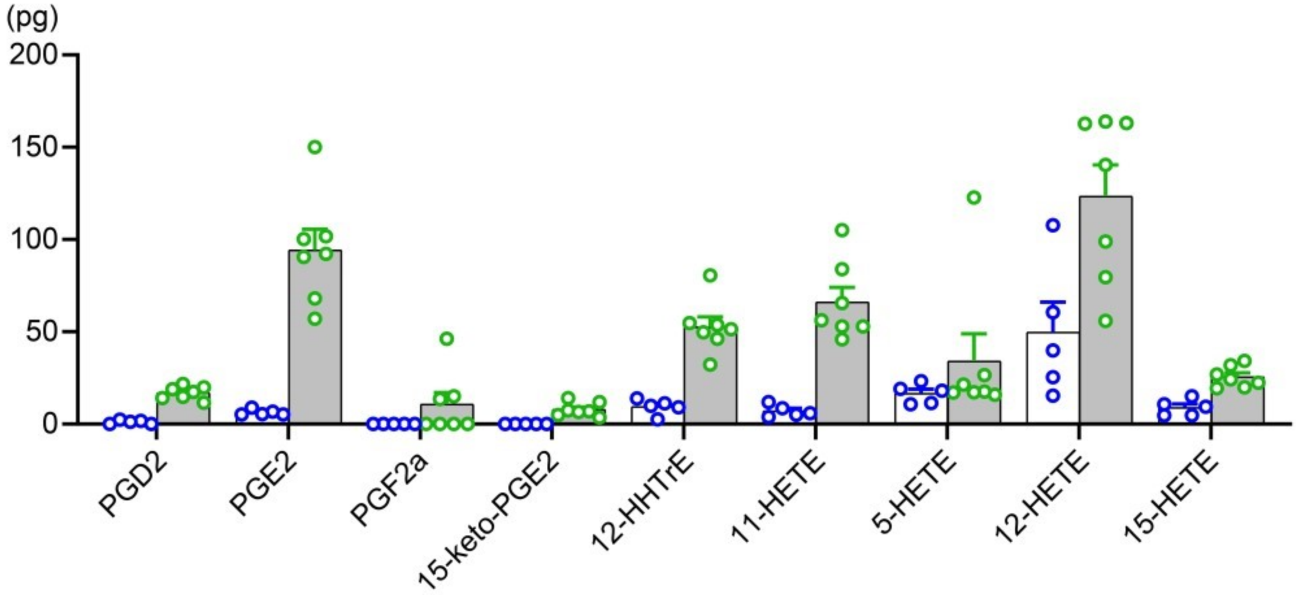

Supplementary Figure 2. Effects of smoking exposure on lipid mediators in the bronchoalveolar lavage fluid (BALF) under single smoking exposure. Related to Figure 5. The graph shows a comparative analysis of the amounts of arachidonic acid (AA)-derived metabolites produced by cyclooxygenase (COX), 5-lipoxygenase (LOX), and 12/15-LOX. Vehicle-plus-air group (white bar); Vehicle-plus-smoke group (gray bar).

Original uncropped gels and blots

Figure 2 (E)

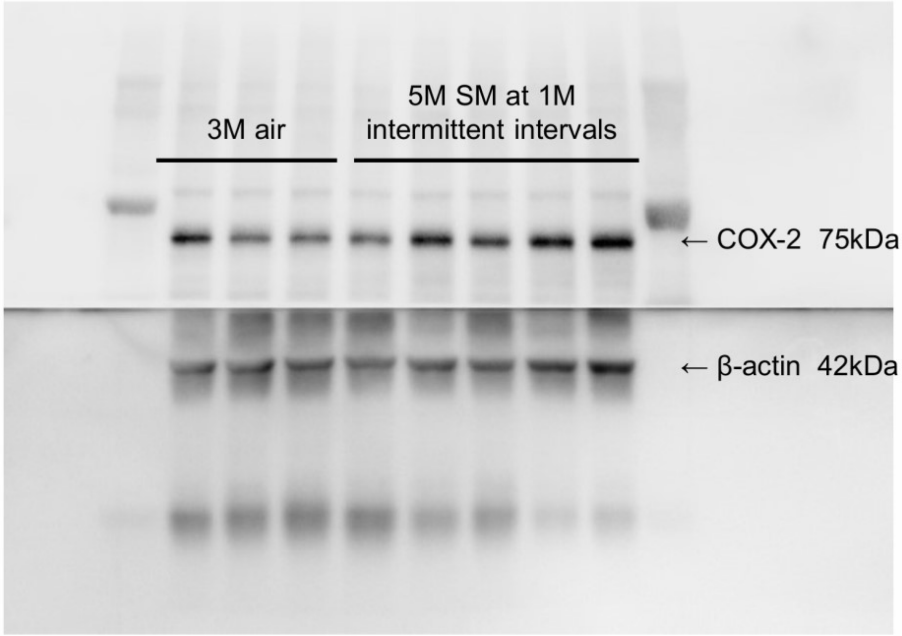

Figure 2 (G)

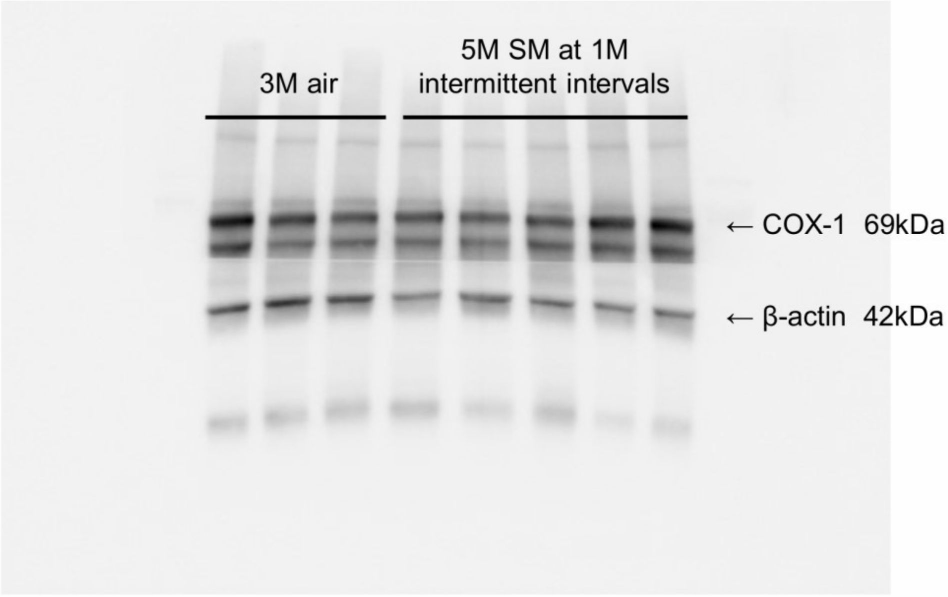

Supplementary Figure 3. Original uncropped gels and blots corresponding to **Figure 2E** and **2G**.
